# Electrotherapy as a Means of Diagnosis in Gynæcology*Read before British Medical Association, London (1895).

**Published:** 1895-10

**Authors:** G. Apostoli

**Affiliations:** Paris


### ELECTROTHERAPY AS A MEANS OF DIAGNOSIS IN GYNÆCOLOGY.*

BY DR. G. APOSTOLI (OF PARIS).

Dr. Apostoli, after a long and thorough trial of his method, has come to the following general conclusions:

1° *The faradic current of tension* (generated by the coil of long and fine wire) applied to the uterine cavity, according to the rules established by Dr. Apostoli, in 1883, relieves, for a longer or shorter time, all ovarian pain of nervous or hysterical origin; but remains powerless, or nearly so, in cases of ovarian pain caused by inflammatory lesion of the peri-uterine tissue, or of the appendages.

2° The same faradic current is therefore useful in diagnosis inasmuch as it helps us to distinguish the nature of so-called ovarian pain, and to determine rapidly the differential diagnosis between hysterical and inflammatory ovarian pain. Where the two kinds of pain exist in the same patient, we are helped to understand their nature by the fact that the one is relieved, and the other is not.

3° If, then, the curative effect of the faradic current clears up or rectifies a doubtful diagnosis, it protects us, at the same time, from undertaking a useless operation.

On the other hand, if the same faradic current proves ineffective, the lesion being inflammatory, we are led to resort to a supplementary galvanic treatment, or to a surgical operation, sooner or later.

4° *The constant galvanic current*, applied to the uterine cavity in doses gradually increasing from 50 to 120 milliamperes, according to the rules published by Dr. Apostoli, in 1884, and bearing in mind the individual susceptibility and tolerance, will be almost always supported without much pain during the seance, and without febrile reaction afterward, if the parts adjacent to the uterus are free from inflammation.

Simple cystic, peri-uterine tumors, which are neither inflamed nor suppurating (such as ovarian cysts and hydrosalpinx), may also show perfect tolerance of the galvanic current.

*Read before British Medical Association, London (1895).

The galvanic current is also sometimes perfectly supported by cases in which the uterus is surrounded by old inflammatory products or exudations no longer pathogenic.

5° There are three classes of cases which should be considered as exceptions to the preceding rule, for they bear the galvanic current more or less badly, though they do not necessarily produce much febrile reaction after the seance. They are:

A.—Certain forms of hysteria.

B.—Fibro-cystic tumors of the uterus.

C.—Enteritis, with false membrane.

It is generally easy to diagnose these cases of intolerance.

6° All acute peri-uterine inflammation (of the pelvic cellular tissues, of the peritoneum, and especially of the appendages) will cause the galvanic current to be badly borne when it passes 40 or 50 milliamperes, and will cause intolerable pain and febrile reaction when carried beyond this intensity.

7° The intolerance for the galvanic current is generally proportionate to the extent and gravity of the lesions referred to, and increases with the intensity of the current employed—especially when it passes 40 or 50 milliamperes.

8° All inflammation of the appendage which is *curable* (*symptomatically at least*) without radical operation, will bear the galvanic current better and better, and there will be a corresponding improvement of the prominent symptoms, such as pain and hemorrhages.

The intolerance noted at the beginning progressively disappears.

9° All grave inflammatory lesions of the appendages, and notably all suppurative processes which are *incurable* (*even symptomatically*) by conservative means, show the same intolerance from the beginning to the end of the treatment which was noticed at first, and which is apt to increase instead of diminish if the treatment is continued.

10° Thus, the simple study of the tolerance or intolerance of the intra-uterine galvanic treatment, and especially of the post-operative pain and fever occurring on the evening of, or the day following the treatment, enables us to make the diagnosis. It also, in 4 or 5 seances, given twice weekly, informs us of the condition of the appendages, of their possible inflammation and its degree, and in this way it lessens the number of laparotomies and exploratory incisions.

11° The same study of the so-called galvanic reactions also informs us rapidly (in 5 or 10 seances) of the curability of these inflammatory lesions which the electric current has demonstrated, and in consequence of this it tells us in one case to abstain from operation while in another it shows an operation to be urged.

12° *En resume*, Gynæcological Electro-Therapeutics, carefully, methodically and patiently applied, instead of beirg opposed to the marvellous progress of surgery, comes to its aid. Independently, in fact, of the great therapeutic service which it renders every day, electricity serves as a *touchstone*; it assists us in diagnosis and thus directly serves the interests of surgery, in one case showing an operation to be useless and dangerous, in another that its necessity is urgent.

Thus many laparotomies, so-called exploratory incisions and mutilations practiced without due deliberation for the relief of rebellious ovarian pain or for lesions of the appendages of uncertain nature, should be, from this time forth, delayed or formally proscribed until all the resources of *faradic sedation* on the one hand and of the *intra-uterine galvanic effect* on the other, have been tried. Experience has abundantly proved these currents to be innocuous, if given with necessary aseptic precautions.

---

DETECTION OF SUGAR IN THE URINE.—Dr. A. R. Elliott (*N. Y. Medical Journal*, July 27th), gives the following formula, devised by himself, for the testing of sugar in the urine:

Solution 1.

Cupric Sulphate, gr. xxvii.

Glycerine (Pure), ʒ iii.

Distilled Water, ʒ iiss.

Liq. Potassæ, ad. ℥ iv.

Dissolve the cupric sulphate in the glycerine and water, and gently heat. When cold, add the liquor potassæ.

Solution 2.—A saturated solution of chemically pure tartaric acid.

These solutions are quite stable, and keep indefinitely.

Into a test tube pour a drachm of the cupric oxide solution. Boil gently over a sp.rit lamp. Then add two or three drops of the tartaric acid solution, and boil again. The urine is now added slowly, drop by drop, until eight drops are added. If no reaction takes place by this time, there is no sugar. The reaction is a yellowish, or reddish, or greenish-gray deposit of suboxide, which is marked and unmistakable. In a few minutes, the reaction deepens.—*Medical World*.

